# Change in demand for health-related undergraduate studies in Spain during 2015–2021: a temporal series study

**DOI:** 10.7717/peerj.16353

**Published:** 2023-11-08

**Authors:** Javier Jerez-Roig, Dyego L. Bezerra de Souza, Irene Cambra-Badii, Jaume-Miquel March-Amengual, Agustí Comella, Montse Masó-Aguado, Anna Ramon-Aribau, Alejandro Luque-Suárez, Lydia Feito Grande, Núria Terribas, Luis Vivanco, Ester Busquets-Alibés

**Affiliations:** 1 Research Group on Methodology, Methods, Models and Outcomes of Health and Social Sciences (M_3_O), Faculty of Health Sciences and Welfare, Center for Health and Social Care Research (CESS), University of Vic-Central University of Catalonia (UVic-UCC), Vic, Spain; 2 Institute for Research and Innovation in Life Sciences and Health in Central Catalonia (IRIS-CC), Vic, Spain; 3 Department of Collective Health, Federal University of Rio Grande do Norte, Natal, Rio Grande do Norte, Brazil; 4 Department of Physiotherapy, University of Málaga, Málaga, Spain; 5 The Biomedical Research Institute of Málaga (IBIMA), Málaga, Spain; 6 Faculty of Medicine, Universidad Complutense de Madrid, Madrid, Spain; 7 Grífols Foundation Chair of Bioethics, Universitat de Vic, Vic, Spain; 8 Platform of Bioethics and Medical Education, Center for Biomedical Research of La Rioja (CIBIR), Logroño, Spain; 9 National Center of Documentation on Bioethics, Rioja Health Foundation (FRS), Logroño, Spain

**Keywords:** Enrollment, COVID-19, Trends, Health, Higher education, Students

## Abstract

**Introduction:**

The expansion of higher education is a worldwide phenomenon. To our knowledge, there are no studies analyzing the trends in demands of enrollment in health-related studies in Spain. Therefore, the objective was to analyze the change in demand (the number of requests for enrollment divided by the number of offered places) for undergraduate health-related studies in Spain during the period 2015–2021 as well as compare the change by region in the pre (2015–2019) and pandemic (2020–2021) period.

**Methods:**

This is an observational (ecological type) study with temporal series analyses using data from public (non-for-profit) higher education institutions from the Integrated University Information System. For the analysis by region, we calculated the demand of all twelve undergraduate health-related degrees and the percentages of change between both periods using the Wilcoxon test. The Joinpoint Regression program was used to analyze the trends in demand for each degree during the 7-year period.

**Results:**

Significant (*p* < 0.001) increases in demand during the pandemic period were observed in all regions. During the pandemic, medicine, biomedicine, nursing, odontology and pharmacy presented a higher demand in comparison with data collected before the pandemic started. In contrast, this pattern was not confirmed in the following cases: physiotherapy, occupational therapy, podiatry, psychology, social work, human nutrition and dietetics. By regions, Navarra, Asturias, and La Rioja presented the most drastic changes. In regions with the biggest number of universities, such as Catalonia, Andalusia and Madrid, the change observed was smaller.

## Introduction

The global proliferation of higher education encompasses the growth of higher education institutions and the widespread enrollment in universities (Adejimi & Nzabalirwa, 2021; Gumport et al., 1997). The democratization of education began in Western Europe and America especially after the Second World War, and it was extended to other continents. This democratization has led to gradual equality of access and opportunity in education (Resnik, 2007), with a notable increase in female enrollment (Wan, 2018). In Spain, the expansion of higher education, especially after the 1990s, is related to the creation of universities in the different regions (Autonomous Communities) and the increase of the educational offer and public funds, including the development of the public scholarship system (López, 2009).

The first universities in Spain date back to the 13th and 15th centuries, and private institutions have existed since the 20th century. After the Spanish civil war, the enrollment of women in universities declined markedly until the arrival of democracy in 1978 (Flecha Garcia, 2011). In 1983, the Educational Reform Bill devolved higher education to the different autonomous communities, promoting higher education (Garcia-Andreu, Acebal Fernandez & Aledo, 2020), and 28 universities were founded in the 1990s (Bricall, 2000). Degree offerings were also expanded, promoting access to higher education even more (Angoitia & Rahona, 2007). In 1999, the Bologna declaration proposed comparable systems between different countries for European university education, although Spain did not include all its proposals in the Organic Law 4/2007, of April 12 (Corominas & Sacristán, 2019). The current structure of university education in Spain was established by the Royal Decree 1393/2007, of October 29, in alignment with the general guidelines of the European Higher Education Area. Students need to request admission and carry out an admission test or an interview in order to be admitted to a university. Nowadays, public higher education comprises 50 universities, while the private higher education sector includes 34 (Ministry of Education and Vocational Training of Spain, 2022). Regarding health-related studies, there are 421 university degrees, 264 from public universities, with 188,553 students (73%), and 157 from private universities, with 69,352 students (27%) (Ministry of Education and Vocational Training of Spain, 2022).

Previous research on higher education enrollment has mainly focused on trends and barriers for women, people with disabilities, racial/ethnic groups, and poverty (Brown et al., 2000; Rothman, Lipset & Nevitte, 2003; Barr & Turner, 2013; Triventi, 2013; Wan, 2018; Alonzo et al., 2019; Ayalon & Mcdossi, 2019), but analyzing the trends in public demand for higher education is also important to study the optimization of financial and human resources (Abankina & Filatova, 2015). The influence of crises and periods of economic expansion has been analyzed over long periods regarding fluctuations in demand (Baum, Kurose & McPherson, 2013; Frisbie & Converso, 2016). In the US, for instance, the rising cost of higher education marks a decreasing trend (Cain et al., 2014).

Beyond fluctuations in university enrollment over long periods, analysis of the effects of specific events, such as 9/11 in the US has also been carried out (Alberts, 2007). In this case, the analysis focused on the influence of these attacks on the restrictions of mobility and acceptance of international students. The COVID-19 pandemic, declared a global public health emergency in March 2020 (World Health Organization (WHO), 2020), can be considered to be one of the major health and socioeconomic crisis of the decade. It led to, especially during 2020, an overburdening of local health systems and the need for lockdowns and other restrictive measures to control the spread of disease. Since COVID-19 began to spread in Europe, Spain has been one of the most affected countries, with a special impact on healthcare workers (Rodríguez-Rey, Garrido-Hernansaiz & Bueno-Guerra, 2020). As of 2021, Spain ranked as the third European country with the highest number of COVID-19-related fatalities and was also among the first in terms of the strictness of its lockdown measures (Tejedor et al., 2021).

In the context of the COVID-19 pandemic and the university environment, one of the primary areas of research focus pertained to its impact on the mental health of college students (Abbas et al., 2020; Byrnes et al., 2020; Choi et al., 2020; Sundarasen et al., 2020; Wang et al., 2020, 2022b; Fu et al., 2021; King et al., 2022). Concurrently, new research studies emerged in relation to the career paths of undergraduate health science students, examining how the pandemic affected their post-graduation career plans (Wang et al., 2020, 2022a; Deng et al., 2021; Krier et al., 2022), and the challenges of online education (Farooq, Rathore & Mansoor, 2020; Li et al., 2021; Mirzaian & Franson, 2021).

To the best of our knowledge, there have been no studies conducted that consider the impact of the COVID-19 pandemic on the demand for undergraduate studies in Spain. Previous research on the demand for undergraduate studies in Spain has focused on inclusion and barriers to access (Márquez & Melero Aguilar, 2020), the influence of socioeconomic and cultural background such as economic level and parental education (Mora, 1997; López-Valcárcel & Quintana, 1998), family background and employment expectations (Albert, 2000), and local labor market conditions (Petrongolo & San Segundo, 2002).

Beyond COVID-19 pandemic, the trend of expanding enrollment in universities is also observed in health sciences degrees. In the US, since 2008, the number of bachelor’s degrees in health professions and related programs has increased by 67% (Snyder, de Brey & Dillow, 2018). One previous research study focused on enrollments in kinesiology in the US and concluded that the growth in undergraduate kinesiology enrollments reflects an increase in student interest in the health professions (Bassett et al., 2018). To our knowledge, there are no studies about the trends in demand for health-related bachelor studies in Spain.

The current needs of society and the perception of a worldwide health crisis that continues may have increased interest in pursuing university degrees in the area of health and social care. We hypothesized that there has been an increase in the demand for first-year enrollment of Spanish university degrees in health sciences since 2020, with the start of the pandemic. The objective of this study was to analyze the trends in demand for first-year undergraduate health-related degrees in Spanish public higher education institutions during the period 2015–2021. A secondary objective was to analyze the change of overall demand for the 12 undergraduate health-related studies in the different Autonomous Communities of Spain, comparing the pre-pandemic (2015–2019) and pandemic (2020–2021) periods.

## Materials and Methods

This is an observational (ecological type) study with temporal series analyses using open access data from the General Secretariat for Universities of the Ministry of Universities (Spanish Government) (Spanish Integrated System of Universities Information, 2022). The analyses were made with the database that contains the pre-registration and registration data for the 47 public Universities in Spain. The data used were the offered places, the enrollment of new students by pre-registration, and the pre-registered students in first option. New enrollment data was available only for private universities, so it was not possible to use the indicator of demand in the study. Since this study uses publicly available secondary data, ethical approval was not necessary. The datasets generated and/or analyzed during the current study are available in the web page of the Ministry of Universities (Ministry of Universities of Spain, 2022).

We collected information on the number of offered places at Spanish public universities for health-related undergraduate studies (biomedicine, pharmacy, medicine, odontology, nursing, physiotherapy, occupational therapy, podiatry, psychology, social work, human nutrition and dietetics, biomedical and bioinformatics engineering) and demand. The latter was calculated from the number of requests for enrollment divided by the number of offered places for each study and year. For the analysis by Spanish Autonomous Communities, we calculated the demand of all twelve undergraduate health-related degrees during the pre-pandemic (2015–2019) and pandemic (2020–2021) periods. Also, the percentages of change between both periods were calculated for each region. This methodology was based on the work done by Alberts (2007). We tested normality of quantitative data (demand) for the 17 Autonomous Communities through the Shapiro-Wilk test. Since *p* values for the first and second periods were 0.018 and 0.154, respectively, we used the Wilcoxon test to analyze changes in the demand between the regions.

For the specific analysis by undergraduate degree, we first conducted a descriptive analysis of enrollment indicators showing absolute and relative frequencies. Second, analyses of enrollment trends were carried out by calculating indicators for each term/year through the Joinpoint Regression Program software version 4.7.0.0. Seven years were considered: five pre-pandemic years (2015, 2016, 2017, 2018 and 2019) and two after the COVID-19 outbreak (2020 and 2021). The purpose was to determine whether there was a statistically significant joinpoint associated with the pandemic outbreak for each undergraduate degree. Joinpoint regression is a statistical modeling technique that attempts to explain the relationship between two variables through regression lines. The points where these lines join are called joinpoints. This model assumes a linear trend between these points and shares the same assumptions as linear regression, except for homoscedasticity and autocorrelation. However, the software allows for the incorporation of these conditions when violated, meaning it fits a weighted regression model (Kim et al., 2000). Joinpoint analysis identifies the moment in which changes in trends occur and calculates the annual percentage change (APC) in each segment. The analysis starts with a minimum number of joinpoints and contrasts whether one or more are significant to the model (Kim et al., 2000).

## Results

### Demand by regions

**Figure 1:**
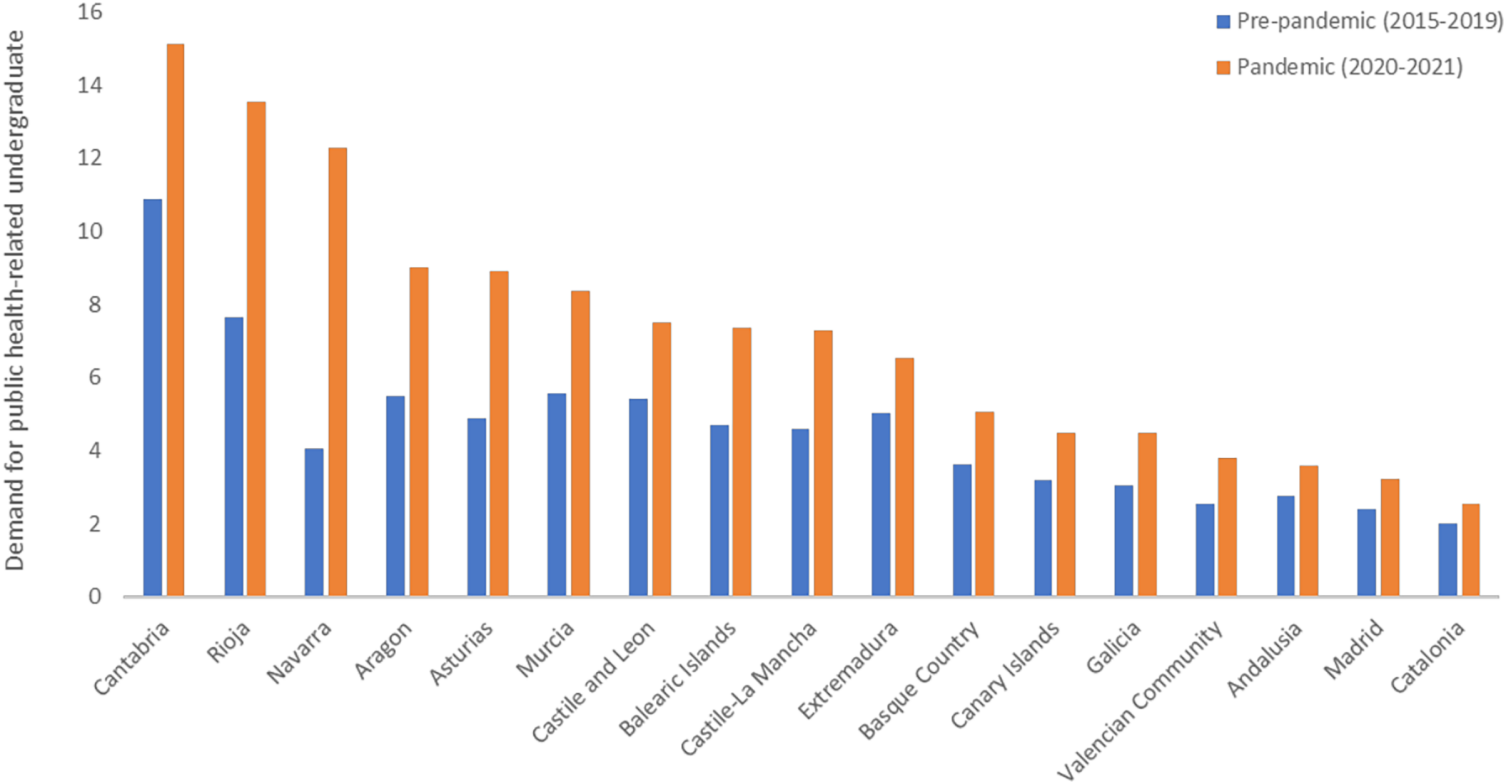
Demand for 12 undergraduate health-related degrees in Spanish public higher education institutions during the pre-pandemic (2015–2019) and pandemic (2020–2021).

Figure 1 shows the demand for public health-related undergraduate degrees in Spain, comparing the pre-pandemic (2015–2019) and pandemic (2020–2021) periods. Overall, it can be observed that the demand varies between 2 and 15 (number of requests for enrollment/number of offered places) during the whole period, being higher in the pandemic period in all 17 Spanish regions. The regions with the overall highest demand of health-related undergraduate degrees were Cantabria, La Rioja and Navarra. On the other hand, Catalonia, Madrid and Andalusia present the lowest demand, with between approximately two and three requests for enrollment per place.

Table 1 contains specific values of demand for each region. The increases in the demand (% of change) comparing the pre-pandemic and pandemic period were statistically significant (*p* < 0.001) according to the Wilcoxon test. The highest increase (comparing the pre-pandemic and pandemic periods) was identified in Navarra where the demand doubled, followed by Asturias and La Rioja. On the other hand, the regions with the lowest percentage of change were Catalonia, Andalusia and Extremadura.

**Table 1:** Demand for undergraduate health-related degrees in public higher education institutions in Spain during the pre-pandemic and pandemic periods and percentage of change between both periods.

**Region**	**Pre-pandemic**	**Pandemic**	**% Change**
Navarra	4.1	12.3	203
Asturias	4.9	8.9	83
La Rioja	7.6	13.5	77
Aragon	5.5	9.0	64
Castile La Mancha	4.6	7.3	59
Balearic Islands	4.7	7.4	56
Murcia	5.6	8.4	50
Valencian Community	2.6	3.8	49
Galicia	3.1	4.6	47
Canary Islands	3.2	4.5	41
Cantabria	10.9	15.1	39
Castile and Leon	5.4	7.5	39
Basque Country	3.6	5.1	39
Madrid	2.4	3.2	34
Extremadura	5.0	6.5	30
Andalusia	2.8	3.1	30
Catalonia	2.0	2.5	27

### Demand by degree

**Figure 2:**
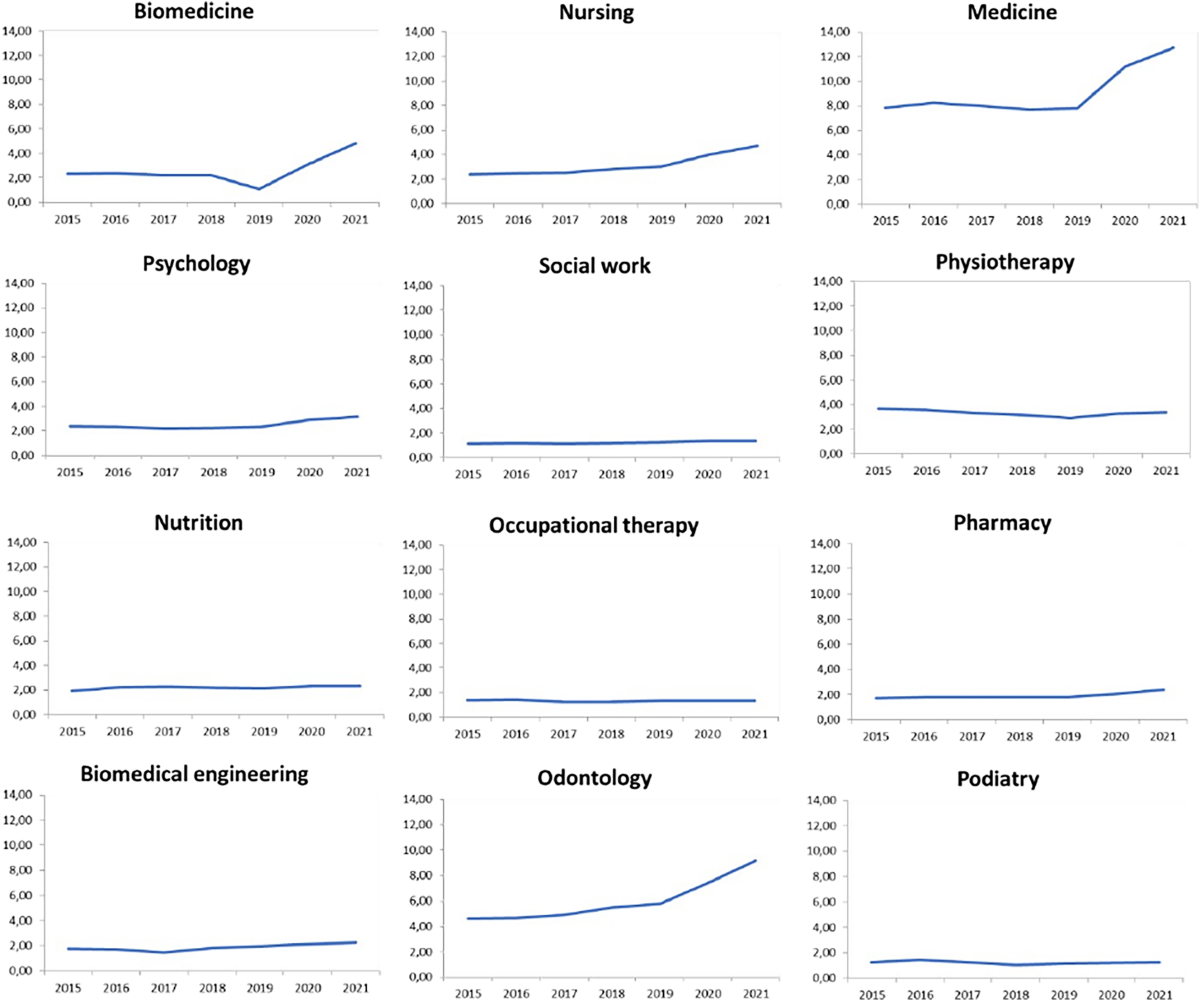
Trends of demand for undergraduate health-related studies in public higher education institutions of Spain during the period 2015–2021.

Figure 2 shows the enrollment trends in the different undergraduate health-related degrees in Spain by year (for the period 2015–2021). Medicine was the most in-demand degree during the whole period, increasing from approximately eight applications per official place in 2015 to more than 11 in 2021. The next most in-demand degrees were odontology (from approximately 4.5 applications/place to 7.5), nursing (from 2.5 application/place to 4) and physiotherapy (from 3.5 applications/place to 3). The rest of the degrees had a demand lower than three applications per place during the whole period. It is worth noting the increasing trend of degrees such as biomedicine and psychology, among others, which are further analyzed in Table 2.

**Table 2:** Enrollment trends of health-related undergraduate degrees in Spanish public higher education institutions during the period 2015–2021.

**Degree**	**Mean (demand)**	**Standard deviation**	**APC1**	**95% CI**	**Joinpoint**	**APC2**	**95% CI**
Biomedicine	2.60	1.14	−13.6*	[−17.5 to −9.4]	2019	84.7*	[59.2–114.2]
Nursing	3.12	0.88	6.8*	[0.6–13.4]	2019	26.4*	[4.5–52.9]
Medicine	9.05	2.03	0.0	[−6.4 to 6.8]	2019	29.0*	[4.8–58.9]
Psychology	2.49	0.37	−3.5	[−15.4 to 9.9]	2018	14.1*	[0.1–30.0]
Social work	1.23	0.09	1.3	[−10.8 to 14.9]	2018	5.2	[−7.2 to 19.4]
Physiotherapy	3.31	0.25	−5.6*	[−8.1 to −3.0]	2019	7.3	[−1.5 to 16.8]
Nutrition	2.19	0.15	6.6	[−14.1 to 32.3]	2017	0.8	[−5.8 to 8.0]
Occupational therapy	1.32	0.08	−0.9	[−4.0 to 2.2]	–	–	–
Pharmacy	1.90	0.24	0.8	[−2.3 to 4.1]	2019	14.9*	[3.9–27.0]
Biomedical engineering	1.89	0.26	−6.1*	[−7.8 to −4.4]	2017	10.4*	[9.7–73.3]
Odontology	5.99	1.70	6.5	[−0.1 to 13.4]	2019	26.9*	[3.9–55.1]
Podiatry	1.21	0.12	−7.2	[−29.4 to 22.0]	2018	3.3	[−21.4 to 35.8]

**Note:**

* Statistically significant (*p* < 0.05).

A significant increase in the demand for enrollment during the pandemic period of 2020–2021 was observed for five undergraduate studies. The highest was in biomedicine (84.7%), followed by medicine (29.0%), odontology (26.9%), nursing (26.4%) and pharmacy (14.9%). In physiotherapy, the increase (7.3%) was not statistically significant. In psychology the increasing trend started 1 year earlier, in 2018, according to the Joinpoint program.

Regarding biomedicine, a significant decreasing trend was observed in the initial period (2015–2019), followed by an increasing trend in 2020–2021. In nursing, a slight increasing trend was identified during the period 2015–2019, followed by a significant increase in 2020–2021. An initial period of stability was observed in medicine, odontology and pharmacy (0.0%, 6.5% and 0.8%, respectively), followed by a significant increase in 2020–2021 (29.0%, 26.9% and 14.9%). In psychology and occupational therapy, an initial period of stability (3.5% and –5.0%, respectively) was observed until year 2018, followed by a significant increasing trend (14.1%) in psychology and a non-significant increase in occupational therapy (3.3%). Physiotherapy showed a significant decrease (−5.6%) during the period 2015–2019, followed by a non-significant increase in 2020–2021. A significant slight increasing trend (3.2%) was observed for social work, with no resulting joinpoint during the 7-year period. Stability was observed for nutrition studies during the whole period (2.4%).

## Discussion

This study aimed at analyzing the trends of demand for health-related undergraduate degrees in Spanish public higher education institutions during the period 2015–2021. Overall, we identified a significant increase in this demand in all 17 Spanish regions, comparing the pre-pandemic and pandemic periods. This increase was higher in a few regions in the Northern part of Spain, like Navarra, La Rioja, Cantabria and Aragon. Most of these regions already had a high demand in the first period, and the interest of potential students to pursue health-related studies seems to have increased even more during the pandemic period. Indeed, Navarra is a region with a tradition in health-related studies, especially medicine, with high demand and admission cut-offs. We had the initial hypothesis that the increase of demand for health-related studies could have been higher in regions most affected at the beginning of the pandemic in Spain. Related to this hypothesis, a study conducted in Spain found greater psychological impact on healthcare workers in regions with an initial higher COVID-19 incidence (*i.e*., Madrid, Castile La Mancha, and Catalonia). However, we observed a lower increase in demand in populated regions with a higher number of inhabitants and universities, such as Catalonia, Madrid, Andalusia and the Basque Country.

With respect to the specific analysis by undergraduate degree, our hypothesis regarding an increase in the number of applications for first-year enrollment since the start of the coronavirus pandemic in 2020 was confirmed for several biomedical (medicine, biomedicine, pharmacy, odontology) and socio-health (nursing, physiotherapy) degrees. Different trends were observed in other disciplines, such as social work, nutrition, occupational therapy and podiatry, where demand remained stable. Overall, our results show an increasing trend in the demand for health-related studies during the period 2015–2021.

After the Francoist dictatorship in Spain, the democratic period has been characterized by an expansion of higher education and decentralization of the administration (including in education) in the different autonomous communities (Flecha Garcia, 2011). Since the 1990s, three stages in the trends of public demand for higher education have been identified. The first period was characterized by increasing public demand, associated with the “educational boom” period (1995–2005). After this, market oversaturation and the inability of higher-income social groups to invest in education at the same level led to a decline in public higher education (2006–2010). Then, since 2011, there has been a maintenance of public demand and a restructuring of higher education institutions. After this third phase, there has been a need for a transition for a more efficient higher education system (optimizing human and material resources) and to meet the challenge of improving the quality of education and enhancing the competitiveness of graduates in the labor market (Abankina & Filatova, 2015). In Spain, there has been a sustained decline in the birth rate in recent decades, from 15.2 births/1,000 inhabitants in 1980 to 9.8 and 7.2 in 2000 and 2020, respectively (National Statistics Institute of Spain (INE), 2020). This increasing decline in the college-age population, which has been called a “demographic storm” (Grawe, 2018; Pavlov & Katsamakas, 2020), has been identified as a threat to the future of universities from a business point of view (Pavlov & Katsamakas, 2020). Given that most students who enroll in higher education are young people (aged 20 and lower) and that this population segment is in decline, the overall upward trend in the demand for health-related studies identified in our study may be even more consistent.

With the implementation of university degrees in 2007 (after the Bologna Declaration), the offer of university places increased exponentially in the first few years (especially until 2011) and then continued with an approximate annual growth rate of 18% (Corominas & Sacristán, 2019). This expansion has been mainly generated by the private sector. According to the latest official report on higher education in Spain, five autonomous communities have factors significantly higher than the average in relation to the increase in the number of studies: La Rioja (1.48), Madrid (1.47), Catalonia (1.30), Navarra (1.28) and Cantabria (1.26). In La Rioja, Navarra and Cantabria, the increase is mainly due to the expansion of private universities. However, public universities in the abovementioned regions have increased the number of degrees they offer. This is the case of the Public University of Navarra, which has offered medical studies since 2019; the University of La Rioja, which relaunched its nursing studies in 2019, after making important changes; and the University of Cantabria, which launched the degree of biomedical sciences in 2020 during the pandemic. Although the percentage of degrees at private universities in the health sciences is higher than at public universities, the number of places offered continue to be mainly public (67%) (Corominas & Sacristán, 2019).

We identified an upward trend in the demand of biomedical studies (*i.e*., medicine, pharmacy, biomedicine, biomedical engineering). It is possible that the pandemic has increased the interest of people in pursuing this type of studies. As an infectious disease, COVID-19 has highlighted the role of pathologists in the health care system to undertake tasks such as molecular and serological testing (McCloskey et al., 2020). Apart from frontline healthcare workers, scientists, clinical researchers and pharmaceutical companies have all played an important role during the pandemic, and the media has raised social awareness of this (Wong et al., 2021) It is possible that the pandemic has increased utilitarianism in the context of healthcare, *i.e*., some people have been influenced by societal needs and decided to undertake health-related university studies (Southworth & Gleason, 2021).

It is likely that some factors influencing the decision to pursue a healthcare profession, may have been altered during the COVID-19 pandemic, impacting not only currently enrolled undergraduate students but also prospective students aspiring to pursue a degree in a health-related field. Among these factors, some are intrinsic, like personal motivations and humanitarian inclinations (*e.g*., a desire to serve the community and care for others). Sociodemographic aspects, such as gender and socioeconomic status, also can influence this decision. On the other hand, there are extrinsic factors to consider, including employment prospects, financial security, salary expectations, and the breadth of job opportunities within the healthcare field. Additionally, interpersonal factors like familial influence, peer pressure, the prestige associated with certain healthcare roles, and the potential for social recognition can all impact one’s career choice (Goel et al., 2018; Fuente-Vidal et al., 2021). However, it should be noted that this can be considered an ecological study and therefore it is not possible to empirically establish the causes beyond the observed trends. For instance, the identified increase in the demand of several health-related bachelor studies (*e.g*., medicine, biomedicine, nursing) in year 2020 cannot be directly (and/or only) attributed to the social influence of the Pandemic. To further explore this phenomenon, new qualitative and quantitative studies are necessary.

Regarding other limitations of this work, this observational study has analyzed a period of seven points (years) and only two after the pandemic outbreak. It would be interesting to observe the trend over the coming years, *i.e*., how the relationship between the number of offered places and the social demand evolves. It is also worth noting that this study included indicators of enrollment in public universities; data by gender and from private universities are not publicly available in a general database in Spain. However, our analysis included data from most Spanish universities, and we think that the created indicator of demand reflects the social demand for health-related undergraduate studies. Lastly, it should be noted that a few regions have a low number of offered studies, and this could have increased the demand compared to other regions.

As strengths, it should be noted that we used accurate data during the pre-pandemic and pandemic periods. To the best of our knowledge, this is the first study conducting this type of analysis to explore the evolution of the demand of health-related undergraduate studies in Spain.

## Conclusions

Based on the results, it can be concluded that there has been a significant increase in the demand for health-related undergraduate studies in the pandemic period in all regions of Spain. This change was larger in the northern regions of Navarra, Asturias and La Rioja. In the regions with the highest number of higher education institutions, *i.e*., Catalonia, Andalusia and Madrid, the increase was less notable.

An increasing trend from 2020 (the start of the pandemic) was found in the demand for the degrees of medicine, biomedicine, nursing, odontology and pharmacy. For the rest of the degrees, the trends were different and varied. The information provided in this work can be useful to higher education institutions for planning future trends and to continue analyzing trends not only in relation to the number of potential students in a cohort, but also incorporating external factors that may massively influence the incorporation of students into universities. This analysis could also help in the medium-term planning of health sector institutions to see how it might impact the workforce in these disciplines, the importance of which has been highlighted by the pandemic.

## Supplemental Information

10.7717/peerj.16353/supp-1 Supplemental Information 1 Facts and figures of the System Spanish University.

## Additional Information and Declarations

### Data Availability

The following information was supplied regarding data availability: The data is available in the Supplemental File and from the Ministry of Education and Vocational Training: https://www.educacionyfp.gob.es/servicios-al-ciudadano/estadisticas.html. The data was obtained through the Spanish Integrated System of Universities Information.
